# Inhibiting muscle degeneration in rotator cuff tears: integrative biologic approaches targeting regeneration, fibrosis, and metabolism

**DOI:** 10.1093/stcltm/szag023

**Published:** 2026-05-24

**Authors:** Seok Won Chung, Jong Pil Yoon, Sung-Jin Park

**Affiliations:** Department of Orthopaedic Surgery, Center for Shoulder and Elbow Surgery, Konkuk University School of Medicine, Seoul 143-729, Korea; Department of Orthopaedic Surgery, School of Medicine, Kyungpook National University, Daegu 41944, Korea; Department of Orthopaedic Surgery, Center for Shoulder and Elbow Surgery, Konkuk University School of Medicine, Seoul 143-729, Korea

**Keywords:** cell biology, gene expression, induced pluripotent stem cells (iPSCs), interleukin, mesenchymal stem cells (MSCs), mitogen-activated protein kinases (MAPK), reprogramming, retinoic acid

## Abstract

Rotator cuff (RC) tears are commonly associated with progressive muscle atrophy, fatty infiltration, and fibrosis, which often persist despite successful tendon repair and remain major barriers to functional recovery and structural integrity. These degenerative muscle changes are not sufficiently addressed by tendon-focused surgical approaches and contribute significantly to poor clinical outcomes and high retear rates. In response, biologically targeted therapies have emerged to directly modulate the cellular and molecular mechanisms underlying muscle degeneration. Current strategies focus on 3 major approaches: regenerative cell-based therapies that promote muscle regeneration and suppress fibrosis; signaling pathway modulation to disrupt pathological tissue remodeling; and metabolic interventions aimed at restoring mitochondrial function and correcting lipid metabolism. Central to these strategies is the regulation of dysfunctional cellular populations within the muscle microenvironment, particularly those responsible for fibrotic and adipogenic changes. This article is a narrative literature review summarizing recent preclinical and early clinical evidence, incorporating representative quantitative findings, and acknowledging key limitations of the available studies. By integrating these emerging biologic approaches, this review provides an updated perspective on therapeutic strategies for RC muscle degeneration and evaluates their translational potential for future clinical applications.

## Graphical Abstract

**Graphical Abstract:**
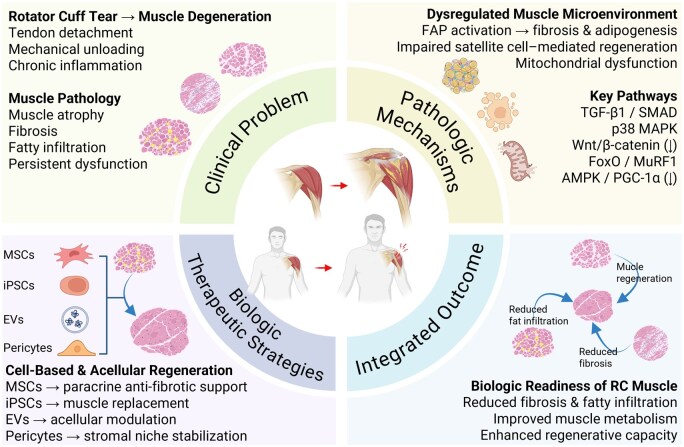

Significance statement Rotator cuff tears are a major cause of shoulder dysfunction, yet current surgical treatments often fail to restore long-term outcomes due to irreversible muscle degeneration, including atrophy, fibrosis, and fatty infiltration. These changes limit strength recovery, increase retear risk, and reduce quality of life after repair. This review highlights emerging biologic strategies cell-based regeneration, targeted signaling modulation, and metabolic reprogramming that actively improve muscle quality beyond tendon repair. By focusing on fibro-adipogenic progenitors as a key therapeutic target, this review outlines integrated biologic approaches with strong translational potential to enhance healing and functional recovery.

## Literature search and scope

To ensure comprehensive coverage, a structured literature search was conducted using PubMed, Scopus, and Web of Science, reviewing studies published between January 2014 and March 2025. Search terms included combinations of rotator cuff, muscle degeneration, fibro-adipogenic progenitors (FAPs), stem cell–based therapies, extracellular vesicles, key signaling pathways (TGF-β1, p38 MAPK, Wnt/β-catenin), and metabolic interventions (β3-adrenergic receptor and GLP-1 receptor agonists). Only English-language preclinical and clinical studies directly related to RC muscle degeneration, FAP biology, or biologic and metabolic interventions were included. Reference lists of key articles were manually screened, while studies limited to tendon pathology without muscle involvement or mechanistic insight were excluded.

## Introduction

Rotator cuff (RC) tears are common in aging populations and are frequently accompanied by progressive muscle degeneration that persists even after successful tendon repair.^1^^,^^2^ Tendon detachment leads to loss of mechanical loading and initiates a degenerative cascade characterized by chronic inflammation, impaired satellite cell activity, and activation of fibro-adipogenic progenitors (FAPs), resulting in muscle atrophy, fibrosis, and fatty infiltration.^3^^,^^4^ Conventional treatments, including physiotherapy, pharmacologic management, and surgical repair, primarily address pain and joint function but have limited capacity to reverse intrinsic muscle degeneration.^3–5^ These limitations have driven interest in biologic strategies that directly target the cellular and molecular mechanisms of RC muscle pathology, broadly encompassing cell-based regeneration, signaling pathway modulation, and metabolic reprogramming.^6–11^ This review summarizes biologic strategies to inhibit RC muscle degeneration by integrating cell-based regeneration, signaling pathway modulation, and metabolic reprogramming.

## Biologic approaches for RC muscle pathology

**Figure 1:**
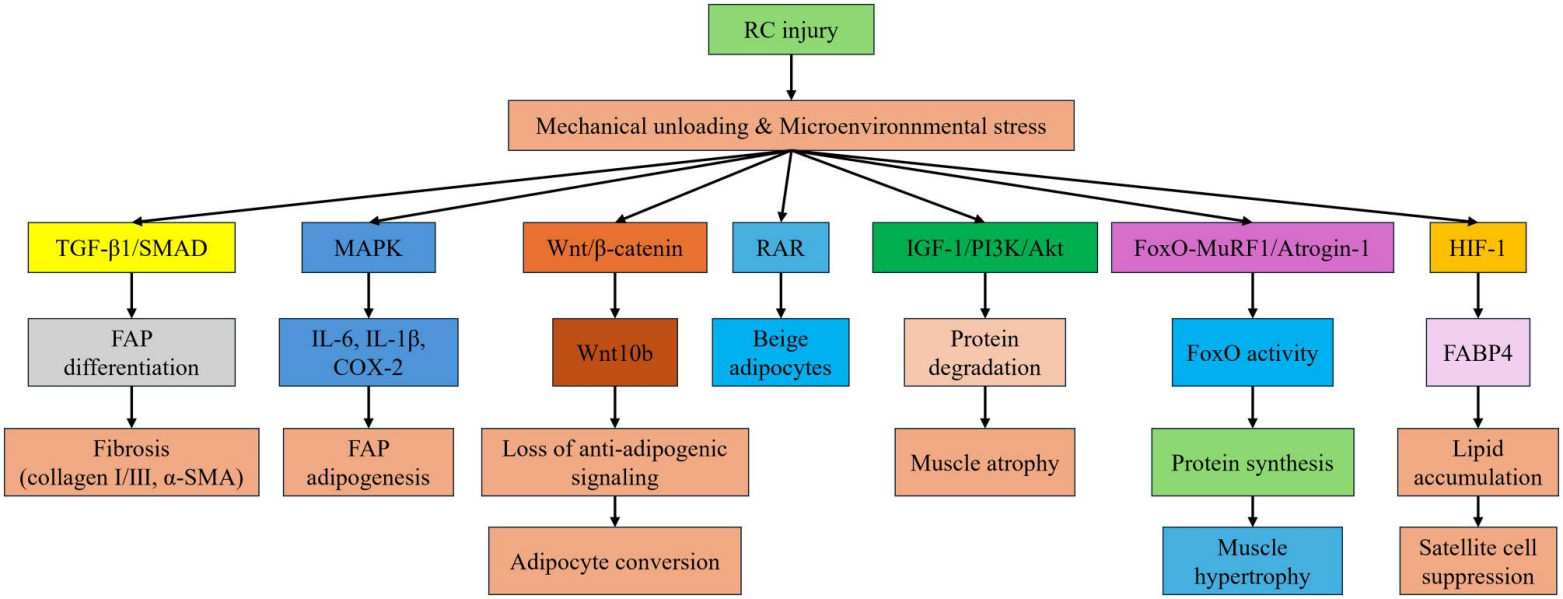
Intracellular signaling pathways contributing to rotator cuff (RC) muscle degeneration following injury-induced loss of mechanical loading following tendon detachment and microenvironmental stress. TGF-β1/SMAD and MAPK signaling promote fibrosis and fibro-adipogenic progenitors (FAPs)-mediated adipogenesis through upregulation of extracellular matrix (ECM) components and inflammatory cytokines. Downregulation of Wnt10b impairs anti-adipogenic signaling, while retinoic acid receptor (RAR) activation favors beige adipocyte formation. Muscle atrophy is driven by FoxO-mediated MuRF1/Atrogin-1 expression, opposed by anabolic like growth factor 1/phosphoinositide 3-kinase (PI3K)/protein kinase B (Akt) signaling. Hypoxia-induced HIF-1 activity increases fatty acid-binding protein 4 (FABP4) expression, promoting lipid accumulation and satellite cell suppression. These integrated molecular circuits collectively impair RC muscle quality and represent targets for therapeutic modulation.

Among these approaches, cell-based regeneration has emerged as a central strategy, leveraging myogenic cells or their paracrine signals to suppress fibrosis and support muscle repair.^6^^,^^10^ Platforms such as stem cell-derived therapies and acellular vesicle-based systems are being actively explored for their regenerative potential.^7^^,^^12^ RC muscle degeneration, characterized by progressive atrophy, fibrosis, and fatty infiltration, remains a major barrier to successful surgical outcomes.^13^^,^^14^ Even after mechanical restoration of the tendon, persistent inflammatory signaling, impaired myogenic regeneration, and FAP activation drive irreversible muscle deterioration (Figure 1).^8^^,^^9^^,^^15^^,^^16^

## Mechanistic basis of biologic therapies for RC muscle regeneration

Biologic therapies exert regenerative and anti-fibrotic effects primarily by modulating cellular behavior and intercellular signaling within the injured RC muscle microenvironment. MSCs act predominantly through paracrine mechanisms rather than direct myogenic differentiation, secreting extracellular vesicles (EVs), cytokines, and growth factors that suppress fibrotic activation, enhance angiogenesis, and support satellite cell-mediated regeneration.^6^^,^^10^^,^^14^ Targeted signaling modulators act on key pathways governing FAP fate. Inhibition of TGF-β1/SMAD and p38 MAPK signaling suppresses myofibroblast differentiation and inflammatory amplification, whereas activation of Wnt/β-catenin signaling restores pro-myogenic programs and restrains adipogenic transcriptional networks.^9^^,^^17^^,^^18^ Together, these cell-based and signaling-targeted strategies converge on a shared mechanistic goal: reprogramming dysregulated cellular populations to restore the balance between degeneration and regeneration in RC muscle.

## Regenerative cell-based platforms: MSCs, iPSCs, and EVs

**Figure 2:**
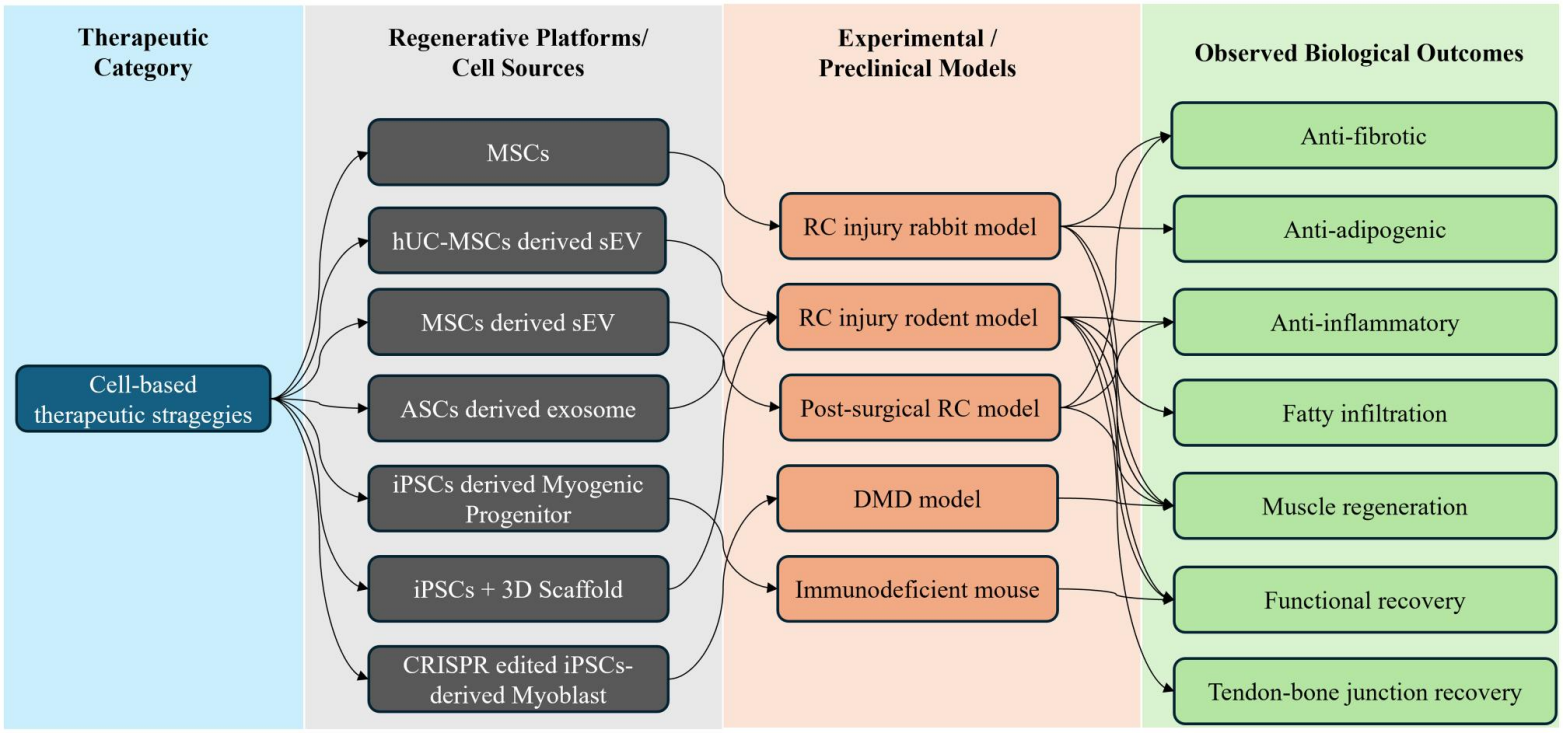
Overview of cell-based therapeutic strategies targeting RC injury. A comprehensive schematic summarizing current cell-based therapeutic strategies targeting the multifaceted pathological changes associated with RC injury. These approaches address not only intramuscular degeneration but also tendon-bone junction defects, inflammation, fibrosis, and fatty infiltration. A wide spectrum of preclinical models including rabbit, rodent, DMD models, and immunodeficient mice has been utilized to evaluate the efficacy of various regenerative interventions. 3D, three-dimensional; ASC, adipose-derived stem cell; DMD, Duchenne muscular dystrophy; hUC-MSC, human umbilical cord–derived mesenchymal stem cell; iPSC, induced pluripotent stem cell; MSC, mesenchymal stem cell; RC, rotator cuff; sEV, small extracellular vesicle.

Regenerative cell-based strategies aim to restore RC muscle structure and function by providing myogenic cells, paracrine support, or regenerative biomolecules that counter fibro-adipogenic remodeling. These approaches primarily target the loss of contractile tissue and pathological stromal activation characteristic of chronic RC degeneration (Figure 2). MSCs derived from bone marrow, adipose tissue, or umbilical cord are among the most extensively studied platforms. Rather than direct myogenic differentiation, MSCs act predominantly through paracrine and immunomodulatory mechanisms, secreting growth factors and EVs that suppress fibrosis, enhance angiogenesis, and support satellite cell activity.^6^^,^^10^^,^^14^ Preclinical models demonstrate improved muscle architecture and reduced fibrotic remodeling following MSC delivery,^19^ while early clinical studies suggest structural benefits, including reduced retear rates.^1^^,^^20^ However, variability in cell quality, delivery methods, and host microenvironment limits consistency and scalability.

iPSCs offer a renewable cell source with high myogenic potential. Following differentiation into Pax7^+^ myogenic progenitors, iPSC-derived cells can structurally integrate into injured muscle and restore contractile properties in preclinical RC models, particularly when combined with biomaterial scaffolds.^7^^,^^21^ Translational challenges remain, including tumorigenicity risk, genomic instability, and maturation control, although recent advances in gene editing and scaffold-assisted delivery have begun to mitigate these concerns.

EVs represent an acellular biologic platform that recapitulates key paracrine effects of stem cells while avoiding risks associated with live-cell transplantation. In RC-specific models, human umbilical cord-derived MSC EVs delivered via injectable collagen improved tendon-bone interface healing and reduced fatty degeneration in a chronic RC tear rabbit model,^22^ while purified exosome products enhanced biomechanical strength and collagen organization in a rat supraspinatus repair model.^23^ Collectively, these findings support EVs as a musculoskeletal-relevant, scalable approach for modulating the repair microenvironment in RC disease.

Complementing stem cell-based strategies, Mosich et al. identified a lineage-restricted, non-fibro-adipogenic pericyte population derived from human embryonic stem cells that attenuated muscle degeneration in a chronic RC injury model.^24^ These PDGFR-β^+^PDGFR-α^-^ pericytes resisted fibrotic and adipogenic remodeling, reduced collagen deposition, and limited myofiber atrophy, supporting a niche-stabilizing, paracrine mechanism rather than direct myogenic replacement.

## Targeting intracellular signaling networks to modulate fibrosis and fat degeneration

Unlike cell-based therapies that primarily replenish myogenic cells, signaling pathway modulation directly targets the molecular drivers of fibrosis, fatty infiltration, and atrophy.^17^ Following RC injury, loss of mechanical loading following tendon detachment and microenvironmental stress activate FAPs, shifting them toward profibrotic and adipogenic fates. Central to this process, TGF-β1/SMAD signaling stimulates collagen and α-SMA expression, driving fibrosis, while p38 MAPK amplifies inflammatory cytokines such as interleukin-6 (IL-6) and IL-1β, reinforcing adipogenesis and impairing satellite cell function.^8^^,^^25^ Loss of Wnt/β-catenin signaling, including rapid downregulation of Wnt10b, promotes fibro-adipogenic differentiation and impairs myogenic regeneration. Conversely, activation of this pathway has demonstrated regenerative potential. For example, the small-molecule Wnt activator WAY-316606 enhances myogenic differentiation and suppresses intramuscular adipogenesis in preclinical models, while romosozumab indirectly stimulates Wnt/β-catenin signaling through sclerostin inhibition, highlighting translational relevance.^9^^,^^14^^,^^26^

In parallel, catabolic forkhead box O (FoxO)-MuRF1/Atrogin-1 signaling accelerates proteolysis, counterbalanced by like growth factor 1 (IGF-1)/phosphoinositide 3-kinase (PI3K)/protein kinase B (Akt)—mediated anabolic activity.^14^ Hypoxia further exacerbates degeneration through the HIF-1/fatty acid-binding protein 4 (FABP4) axis, which amplifies lipid accumulation and fibrosis while suppressing satellite cell regeneration.^5^^,^^27^

Collectively, these interconnected pathways orchestrate RC muscle degeneration by promoting fibrosis, adipogenesis, and atrophy while impairing regeneration. Therapeutic modulation of these cascades offers a disease-modifying strategy that complements tendon repair (Table 1).

**Table 1:** Therapeutic modulation of key intracellular signaling pathways in rotator cuff muscle degeneration.

Pathway	Pathologic role	Representative modulators	Mechanistic effect
**TGF-β1/SMAD**	Fibrosis via alpha-smooth muscle actin (α-SMA) and collagen I expression	Galunisertib, LY2157299	Inhibits fibroblast activation and extracellular matrix (ECM) deposition
**p38 MAPK**	Inflammatory fibrosis and adipogenic activation	Losmapimod, SB203580	Reduces lipid accumulation and collagen deposition
**Wnt/β-catenin**	Downregulation promotes fibro-adipogenic progenitors (FAPs) adipogenesis and muscle atrophy	Wnt10b agonists, romosozumab	Enhances myogenic signaling and suppresses proliferator-activated receptor gamma (PPARγ) expression
**Retinoic acid receptor (RAR)**	Regulates FAPs adipogenic differentiation	Am80 (tamibarotene)	Induces beige adipocyte phenotype and reduces fat infiltration
**Hypoxia-inducible factor-1 alpha/FABP4**	Hypoxia-induced lipid accumulation and fibrosis	Fatty acid-binding protein 4 (FABP4) inhibitors	Decreases fatty infiltration and improves muscle regeneration
**Forkhead box O/MuRF1**	Catabolic proteolysis and myofiber atrophy	GLP-1RAs, β3-AR agonists	Suppresses proteolysis and enhances mitochondrial biogenesis

## Metabolic reprogramming and lipid remodeling

Metabolic dysfunction is a critical driver of RC muscle degeneration, extending beyond fibrotic remodeling and catabolic signaling.^26^ Loss of mechanical loading leads to mitochondrial fragmentation, impaired oxidative phosphorylation, and ROS accumulation, shifting energy metabolism from fatty acid oxidation toward lipid storage.^10^^,^^28^ FAPs are central mediators of this maladaptive lipid remodeling, undergoing adipogenic differentiation under disrupted Wnt/β-catenin signaling and profibrotic cascades such as TGF-β1/SMAD, p38 MAPK, and HIF-1/FABP4.^2^^,^^29^

Hypoxia further accelerates adipogenesis through HIF-1/FABP4-driven lipid storage and suppression of oxidative metabolism, creating a feed-forward loop with mitochondrial dysfunction that culminates in fibrofatty degeneration.^30^ Chronic inflammation and oxidative stress compound this failure by impairing satellite cell function and activating the FoxO-MuRF1/Atrogin-1 proteolytic pathway, leading to persistent atrophy.^31^ Ultimately, pathological metabolic remodeling degrades muscle architecture and function, highlighting a key therapeutic target for restoring RC muscle quality.^31^

## Translational progress and research landscape in biologic therapeutics for RC muscle

### Preclinical advances in cell-based therapies

Preclinical studies have advanced cell-based strategies from bulk transplantation to more targeted, safer modalities. In a rat model, Yea et al. demonstrated that umbilical cord–derived MSCs significantly improved healing of full-thickness RC tendon defects, with enhanced collagen organization and reduced glycosaminoglycan accumulation.^19^ While the primary outcomes focused on tendon repair, histological findings also indicated reduced intramuscular fibrosis and improved tissue quality near the myotendinous junction, suggesting partial benefit to RC muscle integrity.

Zou et al. conducted a systematic review of 46 preclinical studies examining exosome-based therapies for tendon and tendon-to-bone interface healing.^32^ Although these models provide indirect insight into the muscle environment, direct evidence of RC muscle regeneration was limited. The findings nonetheless support the translational relevance of exosome-mediated anti-inflammatory and extracellular matrix (ECM)-modulating effects across musculoskeletal tissues (Table 2).

**Table 2:** Summary of preclinical cell-based interventions for rotator cuff (RC) muscle degeneration.

Study	Model/intervention	Therapeutic agent	Study design/quality indicators	Key quantitative findings
**Yea et al.**	Rat RC full-thickness tear	Umbilical cord-mesenchymal stem cells (local injection)	Controlled animal study (*n* = 12/group), histologic and biomechanical endpoints	Improved collagen alignment (1.99×), failure load (+55%), reduced glycosaminoglycan (GAG) (–94.7%)
**Wang et al.**	Rat massive rotator cuff tear (MRCT)	Adipose-derived stem cell-derived exosomes	Randomized animal model (*n* = 8/group), blinded histologic scoring	Reduced fat infiltration by 71% (quantified by histologic fat area ratio, *n* = 8 per group, *P* < 0.05), increased centrally nucleated fibers by 2.3-fold
**Zou et al.**	Systematic review (46 preclinical studies)	Exosome-based therapies	Meta-analysis (*n* = 2239 total animals across 4 species)	Improved biomechanical integrity (20%-40%), reduced pro-inflammatory cytokines
**Hamer and Rossi**	Murine RC injury + scaffold	Induced pluripotent stem cell-derived myogenic progenitors	Controlled preclinical model, scaffold-enhanced delivery	Improved myofiber density, restored muscle contractility

To address these issues, Wang et al. employed adipose-derived stem cell exosomes (ASC-Exos) in a massive RC tear model.^33^ ASC-Exos reduced fatty infiltration by 71%, macrophage-driven inflammation by 28%, and enhanced myofiber regeneration, offering a safer, cell-free alternative. Building upon these findings, Zou et al. provided a comprehensive systematic review covering 46 preclinical studies involving exosome-based therapies for tendon and tendon-to-bone interface healing.^32^ Their meta-analysis encompassed 1481 rats, 416 mice, 330 rabbits, and 12 sheep, consolidating evidence that exosomes consistently enhance regenerative outcomes.

Meanwhile, Hamer and Rossi developed induced pluripotent stem cell-derived myogenic progenitors (iMPs) for direct muscle fiber regeneration.^7^ In mouse models, iMPs successfully integrated into native tissue and improved both structure and function. Combining iMPs with biomaterials amplified regenerative outcomes, highlighting their translational promise.

In summary, cell-based approaches have evolved from MSC injections to exosome-based delivery and gene-engineered iPSCs. While MSCs and EVs offer paracrine modulation of degeneration, iPSC-derived progenitors uniquely enable structural muscle regeneration. Despite promising data, concerns around engraftment, tumorigenicity, and scalability warrant further investigation before clinical translation.

### Signal modulation as a therapeutic lever against fibro-at degeneration

Beyond cell-based strategies, targeted modulation of intracellular signaling offers a precise approach to counteract RC muscle fibrosis and fatty degeneration by intervening in upstream molecular pathways.

The transforming TGF-β1/SMAD axis is a primary driver of fibrosis, promoting expression of collagen and α-smooth muscle actin (α-SMA). Studies show heightened SMAD activation in torn RC tissues, supporting the use of ALK5 inhibitors (eg, galunisertib) to reduce ECM deposition. The Wnt/β-catenin pathway, particularly Wnt10b, suppresses adipogenic conversion of FAPs. Its downregulation leads to increased peroxisome proliferator-activated receptor gamma (PPARγ) and CCAAT/enhancer-binding protein alpha (C/EBPα) expression, favoring fat accumulation. Wnt activation restores myogenic signaling and counters intramuscular adipogenesis.^18^

The p38 MAPK pathway amplifies inflammatory fibrosis.^34^ Pharmacologic inhibition (eg, SB203580) reduces lipid infiltration and collagen deposition without impairing contractility, indicating selective anti-degenerative effects. Retinoic acid receptor (RAR) signaling also regulates fat remodeling. Agonists like Am80 significantly reduce intramuscular adipocytes and adipogenic markers, suggesting metabolic reprogramming potential without inducing pathological fibrosis.^35^ Lastly, the hypoxia-inducible factor-1 alpha (HIF-1α)/FABP4 pathway promotes fatty degeneration under ischemia. Inhibition of FABP4 decreases fat infiltration and improves muscle strength, interrupting the hypoxia-adipogenesis feedback loop.^36^

Together, these pathways form an integrated therapeutic framework to redirect FAPs from fibrotic and adipogenic fates toward muscle regeneration. Signal-targeting interventions may synergize with tendon repair to enhance structural and functional recovery.

### Metabolic therapeutics: bridging preclinical success to clinical implementation

RC muscle degeneration is fundamentally metabolic, driven by mitochondrial collapse, impaired lipid oxidation, and FAP adipogenesis. This energy imbalance creates a feedback loop of fatty infiltration, fibrosis, and atrophy.^37^ Post-injury mitochondrial dysfunction and reduced ATP availability promote adipogenic FAP differentiation. Pharmacologic activation of the AMPK/PGC-1α pathway using agents such as AICAR or metformin enhances mitochondrial biogenesis and oxidative metabolism in preclinical models, partially correcting lipid dysregulation.^38^ However, AMPK activation alone is insufficient to fully reverse fatty degeneration, underscoring the need for broader metabolic interventions.^14^^,^^37^

More promising results have come from β3-adrenergic receptor agonists. In clinical trials, mirabegron enhanced brown adipose activity, resting energy expenditure, and insulin sensitivity. Muscle biopsies demonstrated reduced triglycerides, increased oxidative fibers, and elevated PGC-1α expression, reflecting restored mitochondrial metabolism (Figure 2).^29^

GLP-1RAs offer another promising avenue.^11^ Systematic reviews and preclinical data show they improve mitochondrial number, function, and morphology while reducing oxidative stress and proteolysis via inhibition of the FoxO/MuRF1 pathway. They also enhance biogenesis markers and promote satellite cell activity.

Together, β3-agonists and GLP-1RAs reduce fat infiltration by over 45%, restore metabolic homeostasis, and support muscle regeneration.^11^ As both drug classes are FDA-approved for metabolic disorders, they present an accelerated translational pathway for RC therapy. Further work is needed to refine musculoskeletal dosing and confirm long-term safety.^11^^,^^28^

## Translational progress and clinical landscape in biologic therapeutics for RC muscle

### Clinical progress in cell-based therapies

Clinical translation of cell-based therapies for RC muscle degeneration has advanced most notably through MSC applications, particularly adipose-derived MSCs (AD-MSCs). In a first-in-human trial, Jo et al. reported that intratendinous injection of autologous adipose-derived MSCs in partial-thickness RC tears resulted in a substantial reduction in shoulder pain and improvement in tendon integrity at 2 years.^20^ However, this was a small, non-randomized trial (*n* < 20 per arm) without a control comparator, and thus the findings should be interpreted as preliminary rather than confirmatory. Similarly, Kim et al. and Cole et al. observed lower retear rates in MSC-augmented repair compared with controls, but functional outcome scores (American Shoulder and Elbow Surgeons, ASES and Constant) showed no significant group differences, indicating structural benefit without consistent functional improvement.^1^^,^^30^

In a randomized trial, Chun et al. confirmed the safety of MSC injection in supraspinatus tears, though no significant clinical benefit was observed over 24 months.^38^ Similarly, Cole et al. evaluated bone marrow aspirate concentrate in RC repair, reporting a retear rate of 18% versus 57% in controls.^30^ Despite superior MRI findings, patient-reported metrics such as (ASES) and single assessment numeric evaluation scores improved comparably across both groups. While these studies establish feasibility and early structural benefits, limitations remain. Outcome variability, lack of standardized protocols, and insufficient mechanistic clarity hinder broader clinical adoption. Rigorous, large-scale trials are warranted to define optimal delivery strategies, dosing, and long-term safety. Regarding iPSCs, translational progress remains preclinical. Advances in generating Pax7+ iPSC-derived MPs show potential for de novo myofiber regeneration and niche repopulation.^39^ However, concerns over tumorigenicity, genomic stability, and maturation fidelity limit immediate clinical use.^7^

Small EVs, especially MSC-derived small EVs, offer a promising acellular alternative.^40^ Preclinical meta-analysis of 46 studies confirms EVs enhance tendon-to-bone healing via anti-inflammatory, pro-angiogenic, and ECM-modulating mechanisms.^40^ Despite strong preclinical support, no human trials have been reported, and translational validation remains a critical next step.

### Translational considerations and practical challenges

Successful clinical translation of biologic therapies for RC muscle degeneration depends on addressing several unresolved practical challenges.^3^^,^^16^^,^^41^ Timing of intervention appears critical, as earlier biologic delivery following tendon detachment may better preserve regenerative signaling, whereas delayed treatment often encounters established fibrofatty remodeling.^5^^,^^14^^,^^16^ Standardization of dosing, delivery methods, and cell preparation remains a major barrier, with current studies varying widely in cell number, delivery vehicles, and manufacturing protocols.^1^^,^^20^^,^^21^

Economic and regulatory feasibility further constrains clinical adoption. Autologous and combination cell-based therapies face challenges related to GMP compliance, scalability, and cost, while allogeneic products offer improved manufacturability but raise immunologic and ethical considerations.^21^^,^^41^

For metabolic therapies, careful risk-benefit assessment is particularly important in older RC patients with cardiovascular or metabolic comorbidities. Agents such as β3-adrenergic receptor agonists and GLP-1 receptor agonists demonstrate systemic metabolic benefits but may require cautious dosing and monitoring due to potential cardiovascular, gastrointestinal, or lean mass-related adverse effects.^11^^,^^27–29^^,^^42^^,^^43^ Together, these translational considerations highlight the need for clinically realistic trial designs that balance efficacy, safety, and feasibility.^16^^,^^21^^,^^41^

### Signal modulation therapies

Targeted modulation of fibrotic and adipogenic signaling pathways is emerging as a promising alternative or adjunct to cell-based interventions for RC muscle degeneration.^30^ These pharmacologic strategies aim to reprogram FAP fate and suppress downstream degeneration.

TGF-β inhibition, most advanced in oncology, has shown antifibrotic efficacy with Galunisertib, which effectively suppresses SMAD2 activity while maintaining a favorable safety profile.^15^^,^^43^ Although musculoskeletal application is untested, its central role in FAP activation highlights strong translational potential. The p38 MAPK pathway has demonstrated muscle-specific clinical impact. The ReDUX4 trial showed that losmapimod reduced fat infiltration and improved muscle function in muscular dystrophy, with good systemic tolerability.^13^ Wnt/β-catenin signaling promotes myogenesis and restricts adipogenesis, and pharmacologic activators (eg, WAY-316606, Romosozumab) have shown regenerative effects in other tissues.^42^ However, risks of ectopic ossification and lack of RC-specific data remain barriers.

Overall, p38 MAPK inhibition currently offers the strongest clinical rationale, while TGF-β and Wnt modulation remain mechanistically attractive but less mature. Key challenges include tissue-specific targeting, dose optimization, and establishing long-term safety.

### Clinical application of metabolic therapeutics

Recent studies highlight metabolic therapeutics as adjuncts to enhance muscle quality in RC degeneration. Two major drug classes, β3-adrenergic receptor agonists and GLP-1RAs, show systemic and tissue-specific benefits, particularly in metabolically vulnerable populations. In a pivotal trial, mirabegron (β3-agonist) improved glucose tolerance, insulin sensitivity, and oxidative muscle fiber content in obese, insulin-resistant adults, with upregulation of mitochondrial markers but without significant weight loss.^27^ Benefits appear to derive mainly from subcutaneous adipose tissue beiging, and safety was favorable, though limitations include small sample size and lack of RC-specific endpoints.

GLP-1RAs such as liraglutide and semaglutide, widely used for diabetes and obesity, demonstrate robust weight loss, cardiometabolic protection, and systemic anti-inflammatory and metabolic effects.^9^^,^^42^ Their safety profile supports repurposing for RC disease, although concerns persist over lean mass loss (up to 40% of weight reduction) and the absence of human data confirming direct mitochondrial benefits in skeletal muscle.^9^ Combining therapy with resistance exercise or protein supplementation may mitigate sarcopenia risk.

Collectively, β3-AR agonists and GLP-1RAs offer complementary mechanisms to reprogram metabolism and suppress fibro-fatty degeneration, representing promising adjuncts to tendon repair.^9^^,^^42^ Rigorous randomized trials with musculoskeletal endpoints remain essential to validate their efficacy and optimize integration into clinical practice.

### Challenges and unresolved barriers in clinical translation of biologic therapies

Despite strong biological rationale, the clinical translation of RC biologics faces significant challenges across cell-based, signaling, and metabolic therapies. For cell-based approaches, MSCs, though generally immunotolerant, may elicit transient inflammatory responses, while iPSCs carry tumorigenicity risks requiring stringent purification and monitoring. Challenges in sourcing, batch variability, and delivery further limit reproducibility and regulatory approval. Signal modulators such as TGF-β and p38 MAPK inhibitors show antifibrotic efficacy but are constrained by systemic toxicity, with adverse cardiac, hepatic, or immune effects reported for agents like Galunisertib and Losmapimod.^8^^,^^15^ Moreover, pleiotropic pathways such as Wnt/β-catenin raise concerns about off-target ossification or oncogenicity. Metabolic therapies benefit from FDA approval in other indications but present musculoskeletal limitations. Mirabegron may induce cardiovascular events in older patients, while GLP-1RAs can cause gastrointestinal symptoms or rare pancreatitis, and their systemic actions complicate tissue targeting.^27^^,^^42^

Additional barriers include translational discordance between rodent models and chronic human RC pathology, as well as regulatory complexity surrounding emerging biologics such as EVs and hybrid constructs. Ensuring safety, manufacturing consistency, and regulatory clarity therefore remains essential for clinical adoption. Despite their promise, biologic therapies continue to face modality-specific challenges across cell-based, signaling, and metabolic approaches, limiting efficacy, safety, and scalability in human populations.

### Comparative assessment of biologic strategies: efficacy, feasibility, and translational readiness

Although cell-based, signaling, and metabolic interventions target distinct biological processes, they share the common goal of improving RC muscle quality and functional recovery. Their relative efficacy and translational feasibility, however, differ substantially.

Cell-based therapies, including MSCs, iPSCs, and EVs, demonstrate the most direct regenerative effects in preclinical models, with improvements in myofiber architecture, fibrosis, and fatty infiltration.^6^^,^^7^^,^^19^^,^^33^ Early clinical studies also suggest structural benefits, such as reduced retear rates when MSCs are applied during RC repair.^1^^,^^20^ Nonetheless, uncertainties regarding engraftment durability, manufacturing scalability, safety, and cost continue to limit widespread clinical adoption.

Targeted signaling modulators offer mechanistic precision by directly regulating pathways that govern FAP fate and pathological remodeling. Inhibition of pro-fibrotic or inflammatory signaling and restoration of pro-myogenic pathways have shown robust effects in preclinical studies.^9^^,^^17^^,^^18^ However, musculoskeletal translation remains early, constrained by concerns regarding systemic toxicity, tissue specificity, and delivery efficiency.

In contrast, metabolic interventions such as β3-adrenergic receptor agonists and GLP-1 receptor agonists indirectly enhance muscle quality by restoring mitochondrial function and lipid metabolism.^11^^,^^28^^,^^29^ Although less tissue-specific, their established safety profiles, systemic delivery, and regulatory approval make them the most immediately translatable strategy, particularly for older RC patients with metabolic comorbidities.

Collectively, these comparisons indicate that while cell-based therapies offer superior regenerative potential and signaling modulators provide mechanistic specificity, metabolic optimization currently represents the most feasible near-term translational approach. Durable recovery, however, will likely require integrated strategies that combine cellular regeneration, pathway modulation, and systemic metabolic reprogramming.

### Limitations of current preclinical and clinical evidence

Despite encouraging preclinical and early clinical findings, biologic therapies for RC muscle degeneration are supported by evidence with important methodological and translational limitations. Preclinical studies predominantly rely on acute injury models in young rodents, which fail to replicate the chronic, age-related, and metabolically compromised environment characteristic of human RC pathology.^19^^,^^32^^,^^33^ In addition, heterogeneity in experimental design, including cell dose, delivery method, and timing of intervention, limits reproducibility and cross-study comparison.

Clinical evidence remains preliminary and constrained by small sample sizes, short follow-up durations, and variable outcome measures. For example, Jo et al. reported substantial pain reduction following adipose-derived MSC injection; however, the study lacked randomization and included fewer than 20 patients per arm.^20^ Similarly, other early trials have demonstrated structural improvements, such as reduced retear rates, without consistent gains in patient-reported functional outcomes.^1^^,^^30^^,^^44^

Variability in primary endpoints further complicates interpretation, as studies emphasize differing combinations of imaging, histologic, and functional measures. To improve translational rigor, future investigations should prioritize large-scale, multicenter randomized controlled trials with standardized protocols, extended follow-up, and integrated structural and functional endpoints, including MRI-based fatty infiltration grading and validated clinical scores (Constant or ASES).

### Future directions for translational research

To advance the translational impact of biologic therapies for RC muscle degeneration, future studies should adopt mechanistically informed designs that bridge preclinical discovery and clinical implementation. Early-phase pilot trials integrating biological and imaging biomarkers may establish proof-of-concept, followed by multicenter randomized controlled trials evaluating multimodal biologic strategies alongside standard surgical care.^21^^,^^36^^,^^41^

Standardization of outcome measures, including validated functional scores (Constant–Murley, ASES), quantitative assessment of fatty infiltration, and sufficient longitudinal follow-up, will be essential to improve reproducibility and clinical relevance.^1^^,^^30^^,^^36^ Future research should also prioritize biologically rational combination strategies and biomarker-driven patient stratification based on FAP heterogeneity to guide clinically actionable studies in RC muscle degeneration.^14^^,^^45^^,^^46^

## Conclusion

RC muscle degeneration results from a complex interplay of muscle atrophy, fibrosis, and fatty infiltration driven by chronic inflammation, dysregulated signaling, and metabolic dysfunction.^3^^,^^4^^,^^14^ This review highlights biologic strategies targeting these mechanisms, including regenerative cell-based platforms, intracellular signaling modulation, and metabolic therapies aimed at restoring mitochondrial health and lipid homeostasis.^6^^,^^7^^,^^9^^,^^11^

Recent transcriptomic and single-cell RNA sequencing studies reveal heterogeneous FAP populations within RC muscle, including pro-fibrotic, pro-adipogenic, and metabolically active subsets that differentially contribute to degeneration and regeneration.^14^^,^^45^^,^^46^ These findings link FAP heterogeneity to key signaling and metabolic pathways such as TGF-β1/SMAD, Wnt/β-catenin, and AMPK/PGC-1α supporting precision modulation of specific FAP subsets rather than global stromal suppression as a therapeutic strategy.^9^^,^^11^^,^^14^

Ultimately, effective treatment of RC muscle degeneration will likely require integrated, multi-modal approaches combining cell-based regeneration, targeted pathway modulation, and systemic metabolic optimization.^11^^,^^16^^,^^41^

## Data Availability

Data sharing is not applicable to this article as no new data were created or analyzed in this study.
